# Supplementary material for: CT abnormalities 3 and 12 months after hospitalization for COVID-19 and association with disease severity: A prospective cohort study
Source: PLoS One. 2024 May 6;19(5):e0302896. doi: 10.1371/journal.pone.0302896 (PMC11073708; doi:10.1371/journal.pone.0302896)
Supplement: S1 File — (DOCX) [file pone.0302896.s001.docx]

**Supplementary file 1**

**Chest CT protocol**

The participating centers used the same volumetric CT protocol across the various CT systems. Tube current settings were adjusted to each patient’s weight and body habitus, but with low dose references at 120 kVp with high pitch, shortest possible rotation time, and field of view according to the body size. CT images were obtained in both supine and prone positions, during breath-holding and deep inspiration. Supplementary expiratory scans were obtained in nine patients. For evaluation of the lung parenchyma and airways multi-planar images were reconstructed with thin (0.9–1.25 mm) section thickness using a high-spatial-frequency (bone) kernel, and a softer kernel with thicker (2–3 mm) sections for mediastinal evaluation. The lungs were examined from apex to base.

**Review and scoring of CT images**

Three thoracic radiologists with at least 10 years of experience reviewed the CT scans from all centers in consensus and in a random order. The observers were blinded to the patient’s clinical conditions and their pulmonary function tests.

The readers registered the presence, extent and distribution of CT features using nomenclature recommended by the Fleischner Society (1). These findings included reticular pattern, GGO, airspace consolidation, parenchymal bands, interlobular septal thickening, and bronchiectasis and/or bronchiolectasis. The presence of mosaicism was also assessed. Reticular pattern, indicative of fibrotic coarseness, was graded as follows: 1, fine intra-lobular fibrosis without evident cysts; 2, predominantly microcystic reticular pattern involving air spaces smaller than or equal to 4 mm in diameter; and 3, a predominantly macrocystic reticular pattern with air spaces larger than 4 mm in diameter, typically corresponding to honeycombing. If ground-glass opacification was superimposed on a reticular pattern, the finding was classified as reticular. These observations were recorded in an electronic database, using a standardized method for evaluation of interstitial lung disease (2).

The distribution and extent of disease was individually assessed in eight zones, four for each lung (above the aortic arch, between the aortic arch and the level of the carina, between the level of the carina and the level of the left inferior pulmonary vein, and below).

The extent of GGO, airspace consolidation and reticular pattern in each zone was assigned a score based on the percentage of lung parenchyma that showed evidence of an abnormality. The score was classified as follows: 0, no involvement; 1, 1 % to 2 % involvement; 2, 3 % to 10 % involvement; 3, 11 % to 60 % involvement. The extent and severity of mosaicism, parenchymal bands and interlobular septal thickening in each zone was scored 0–4 (0, no involvement; 1, minimal; 2, moderate; 3, severe). The extent and severity of bronchiectasis and bronchiolectasis in each zone was scored 0–4 (0, no involvement; 1, bronchial wall thickening without distinct ectasis; 2, mild or moderate; and 3, severe ectasis). An overall score of parenchymal involvement for each patient and each CT feature was derived by summing the scores of the four zones to an ordinal chest CT severity score (CSS) ranging from 0 (no involvement) to 12 (maximum involvement).

For regression analysis and easier visualization of changes in the four CT features of the CSS with a prevalence of abnormalities >20%, we recoded the CSS into an abbreviated CSS (aCSS) scale ranging from 0 to 3 as follows: 0, original CSS score of 0; 1 (mild), original CSS scores of 1–3; 2 (moderate), original CSS scores of 4–7; and 3 (severe), original CSS scores of 8–12.

**References**

1. Hansell DM, Bankier AA, MacMahon H, McLoud TC, Muller NL, Remy J. Fleischner Society: glossary of terms for thoracic imaging. Radiology. 2008;246(3):697-722.

2. Soyseth V, Aalokken TM, Mynarek G, Naalsund A, Strom EH, Scott H, et al. Diagnosis of biopsy verified usual interstitial pneumonia by computed tomography. Respir Med. 2015;109(7):897-903.
