# Supplementary material for: CT abnormalities 3 and 12 months after hospitalization for COVID-19 and association with disease severity: A prospective cohort study
Source: PLoS One. 2024 May 6;19(5):e0302896. doi: 10.1371/journal.pone.0302896 (PMC11073708; doi:10.1371/journal.pone.0302896)
Supplement: Attachment — Submitted filename: rebuttal_letter_plospone_rev1_18mar2024.docx [file pone.0302896.s002.docx]

**Response to editor’s and reviewer’s comments**

**Academic editor**

1. Please ensure that your manuscript meets PLOS ONE's style requirements

**Done.**

2. Thank you for stating the following in the Competing Interests section:
   "Haseem Ashraf reports grant from Boehringer-Ingelheim, Norway. Knut Stavem reports personal fees from UCB Pharma and MSD Norway, unrelated to this study."
   We note that you received funding from a commercial source: UCB Pharma and MSD Norway
   Please provide an amended Competing Interests Statement that explicitly states this commercial funder, along with any other relevant declarations relating to employment, consultancy, patents, products in development, marketed products, etc.

**This has been clarified below and the competing Interests Statement has been amended, as requested.**

**Dr. Haseem Ashraf received an unrestricted grant from Boehringer-Ingelheim, Norway to fund some of the Chest CT examinations in the study. The sponsor had no role or no influence on the study, analyses or interpretation of the findings. Prof. Stavem received grants for consulting assignments completely unrelated to this study (UCB Pharma: writing a report, which was later published in an abbreviated form (Stavem K. Epub 2021/08/27. doi: 10.1080/20016689.2021.1964792. PubMed PMID: 34434534) and MSD Norway: input to a protocol for a study of chronic cough in the general population.**

3. We noted in your submission details that a portion of your manuscript may have been presented or published elsewhere:

   "Yes. The cohort has been presented before, including follow up at 3 and 12 months.
   Crude prevalence of radiology findings has been presented along with lung function/gas diffusion findings, but these prevalences are not presented/repeated in the present publication. The paper focuses on ordinal scores of CT images, including comprehensive analysis of these scores."

   Please clarify whether this [conference proceeding or publication] was peer-reviewed and formally published. If this work was previously peer-reviewed and published, in the cover letter please provide the reason that this work does not constitute dual publication and should be included in the current manuscript.

**The cohort has been presented in several publications, but with emphasis on other outcomes. The objectives, analyses and methodology presented in the current report are original and have not been previously published. Hence, we think the present publication has a different focus and a different analysis that conveys additional and important information to the scientific literature on consequences of COVID-19. The present paper focuses in detail on changes in radiological findings, using ordinal scores and analyses that better use the information in the data, in contrast to a crude analysis on dichotomized outcomes in the previous paper referenced below.**

**The prevalence of dichotomized radiology scores (yes/no) has previously been presented in a peer-reviewed paper (reference #17, revised manuscript). In that paper, we also presented the association of crude, dichotomized CT findings with lung function and gas diffusion. Therefore, this is not repeated here.**

4. Please include captions for your Supporting Information files at the end of your manuscript, and update any in text citations to match accordingly. Please see our Supporting Information guidelines for more information: [http://journals.plos.org/plosone/s/supporting-information](https://mail.uio.no/owa/redir.aspx?REF=2VUSPtYmA6radH6ZIHG-AVA-GMQmjSysDt5NY0EvP1Wnrt4rnD_cCAFodHRwOi8vam91cm5hbHMucGxvcy5vcmcvcGxvc29uZS9zL3N1cHBvcnRpbmctaW5mb3JtYXRpb24.).

**Done.**

5. Please review your reference list to ensure that it is complete and correct. If you have cited papers that have been retracted, please include the rationale for doing so in the manuscript text, or remove these references and replace them with relevant current references. Any changes to the reference list should be mentioned in the rebuttal letter that accompanies your revised manuscript. If you need to cite a retracted article, indicate the article’s retracted status in the References list and also include a citation and full reference for the retraction notice.

**Done.**

**Reviewer#1**

It provides however no information around lung function; patients assessment of breathing and work capacity or physiological measures of the lung function. The main weakness is the lack of information around the association to lung function and even more important as well as patients assessed outcome.

**The objective of the current manuscript was to determine the detailed changes in radiological findings, using detailed ordinal scores and analyses that exploit the information in the data, in contrast to simplified analyses on dichotomized outcomes. Data on lung function, and gas diffusion have previously been presented, and we think more detailed analysis of this is beyond the scope of the present analysis. Therefore, this not been repeated here.**

**However, we have added a comment on this in the Discussion (line 310-315):**

***We have previously shown that there was an improvement in total lung capacity and diffusion capacity of the lungs for carbon monoxide (D_LCO_) from 3 to 12 month after hospitalization for COVID-19, and that there was little association of any fibrotic-like finding on chest-CT with D_LCO_ or modified Medical Research Council dyspnea scores after 12 months in multivariable analysis [17]. Therefore, we have not repeated similar analyses with ordinal radiology scores.***

Some questions that I see could be of value to clarify;
1) How many of the patients were provided steroids, and dose of steroids if given.

**At the time of hospitalization of the patients included in the study (until 1 June 2020; the first wave of COVID-19), steroid treatment for the most severe patients was not standard treatment. Also, patients were treated on a compassionate care basis and subjected to the local standards at each hospital, and not standardized across hospitals.**

**Among those with Chest CT at 3 and 12 months (n=126), the use of steroids was as follows (included in the revised manuscript, lines 161-167):**

***Among patients with chest CT scans at 3 and 12 months (n=126), 5 patients used steroids prior to hospitalization, and only an additional 2 patients were prescribed steroids during the hospital stay. Furthermore, 4 patients received interleukin-1 receptor antagonist (anakinra) and 1 received an infusion with normal human IgG antibodies. In total, 8 of 126 patients were prescribed antibiotics for suspected secondary pulmonary- or other infection. Information on oxygen use was not available at discharge. However, none of the patients used supplementary oxygen at the 12-month follow-up.***

*2) How many of patients experienced any kind further lung infection during the follow-up period, bronchitis, pneumonia, re-infection with Covid or influenza*

**During hospitalization, 8 patients received antibiotics for suspected secondary pulmonary or other infections. We have added a comment on this in the Result section of the revised manuscript (see above). Unfortunately, we do not have information on antibiotic used during the follow-up period.**

3) Where any of the patients included treated with inhaled steroids or other inhaled medications during the follow-up period

**Prior to the COVID-19 related hospitalization, only 13 patients had a known diagnosis of COPD, which could lead to a prescription of inhaled steroids on a regular basis. As mentioned above, only 5 patients used systemic steroids on a regular basis prior to inclusion.**

4) Where any of the patients on systemic steroids e.g. for arthritis

**Please see the responses to comment 1 and 3 above.**

5) How many of the patients were on oxygen therapy after discharge and for how long time.

**Unfortunately, we do not have information on oxygen use at discharge. There were no oxygen users at the 12-month follow-up visit. This has been added to the Results section of the manuscript (line 161-167, see comment 1 above).**
